# Fighting Doping in Elite Sports: Blood for All Tests!

**DOI:** 10.3389/fspor.2019.00030

**Published:** 2019-09-20

**Authors:** Raphael Faiss, Jonas Saugy, Martial Saugy

**Affiliations:** REDs, Research and Expertise in Antidoping Sciences, University of Lausanne, Lausanne, Switzerland

**Keywords:** doping, steroids, testing, urine, serum, hematological passport

## Abstract

In the fight against doping, detection of doping substances in biological matrices is paramount. Analytical possibilities have evolved and sanctioning a doping scenario by detecting forbidden bioactive compounds circulating unmodified in blood is nowadays very attractive. In addition, the World Anti-Doping Agency (WADA) introduced the Athlete Biological Passport (ABP) a decade ago as a new paradigm inferring the use of prohibited substances or methods through longitudinal profiling, or serial analyses of indirect biomarkers of doping, to be both scientifically and legally robust. After the introduction in 2008 of an hematological module (i.e., based on variations of blood variables) aiming to identify enhancement of oxygen transport and any form of blood transfusion or manipulation, a urinary steroidal module was additionally introduced in 2014 composed of concentrations and ratios of various endogenously produced steroidal hormones. Some evidence tends to discredit steroid profiles obtained from urine analyses to detect the use of endogenous androgenic anabolic steroids (EAAS), when administered exogenously, due to high rates of false negatives with short half-life and topical formulations rendering profile alteration only minimal or equivocal. On the other hand, steroid hormones quantification in blood showed a promising ability to detect testosterone doping and interesting complementarities to the ABP thanks to the most recent analytical techniques (UHPLC-HRMS or/and MS/MS). This perspective article explores the opportunities of blood samples to monitor not only hematological but also steroid profiles in elite athletes.

The definition of doping in sport may be subject to several interpretation. According to the World Anti-Doping Code (WADA, 2018a), antidoping provisions may be considered violated when an athlete uses or attempts to use a prohibited substance or method or when a prohibited substance is detected in the urine or blood. The technical ability of an antidoping laboratory to detect such substance is hence key and nowadays warranted by strict international standards for laboratories and operating guidelines. While testosterone was isolated in 1927 and synthetically produced in 1935 (Kremenik et al., 2006), anabolic androgenic steroids (AASs) were only banned by the International Olympic Committee (IOC) and international sporting federations in 1974 (Gosetti et al., 2013) with a widespread screening of AAS since the 1976 Montreal Summer Olympics (275 drug tests with 12 gas chromatographs capable of screening over 200 banned substances) (Dugal et al., 1980; Kremenik et al., 2007). Urine and later blood collections have increased over time to reach the 322,050 samples analyzed in 2017. This large number is often put in perspective with the 2% rate of official adverse or atypical results in the tests performed in laboratories accredited by the World Anti-Doping Agency (WADA) (WADA, 2018b). This rate shall however be interpreted with care for several reasons. First, some tests may preventively deter doping simply by indicating to the athletes that they may be tested at any time. Second, because drug tests give priority to specificity rather than sensitivity: false-negative results underestimate true doping prevalence because of a lack of sensitivity (Kremenik et al., 2006). In an ideal scenario, laboratories would prospectively define and publicize standard testing procedures for all kind of substances, including unambiguous criteria for concluding positivity, and have the procedures validated in blinded experiments beforehand. Such experiments would define the substance, its dose, methods of delivery, timing of use relative to testing, and variations due to individual differences in metabolism (Berry, 2008). However, antidoping is a forensic science, not a medical one. When screening any sample for putative banned substances, the freedom to set sensitivity and specificity to an appropriate level is restricted in an antidoping context (Sottas et al., 2008a).

In parallel, blood samples are widely collected with more than 125,000 analyses in 2017 conducted mostly in serum or plasma (WADA, 2018b) for erythropoiesis stimulating agents (ESAs), growth hormone (GH), or growth hormone releasing factors (GHRFs). Since the first blood tests carried out at the 1994 Lillehammer Winter Olympic Games, blood analyses became widespread before major cycling events in 1997 (Robinson et al., 2005). The introduction of the hematological module of the ABP and its recognized potential (Robinson et al., 2011; Sottas et al., 2011; Schumacher et al., 2012) have consistently helped to make blood sampling more common and more widely accepted by athletes. A widely held view by antidoping scientists is that blood represents a much better human fluid than urine to establish the dose/effect response of a substance and to get a better biological signature of doping (Saugy et al., 2009).

Since several rapid methods to simultaneously detect numerous doping substances exist (Saugy et al., 2000; Ahrens et al., 2012), urine samples are still preferred to blood not only due to less invasive sampling but also because a slower metabolic rate in urine render their concentration of AASs and metabolites higher (or detectable) there at a given timepoint (Gosetti et al., 2013). Finding an exogenous forbidden substance in an athlete's urine sample may represent the simplest way to lead to a rule violation and sanction. However, doping practices have now evolved to circumvent shortened detection windows in conjunction with the exogenous application of micro-doses of substances already present in the body (e.g., testosterone). In response, WADA introduced the Athlete Biological Passport (ABP) a decade ago as a new paradigm inferring the use of prohibited substances or methods through longitudinal profiling, or serial analyses of indirect biomarkers of doping, to be both scientifically and legally robust (Vernec, 2014). A first hematological module (i.e., based on variations of blood parameters) introduced in December 2008 aimed to identify enhancement of oxygen transport and any form of blood transfusion or manipulation (Sottas et al., 2011). With more than 700 sanctions linked to the ABP monitoring for the last 10 years, this indirect approach may be considered successful (WADA, 2018b). A urinary module was additionally introduced in 2014 to monitor various endogenously produced steroidal hormones (Saugy et al., 2014). The bases of a module were indeed already introduced in 2008 (Sottas et al., 2008b). For instance, this steroidal module measures the concentrations of several glucuroconjugated and free urinary compounds linked to testosterone (T) and its metabolism: T, epitestosterone (E), androsterone (A), etiocholanolone (Etio), 5α-Androstane-3α,17β-diol (5αAdiol) and 5β-Androstane-3α,17β-diol (5βAdiol) and the T/E, A/T, A/Etio, 5αAdiol/5βAdiol, and 5αAdiol/E (Kuuranne et al., 2014; WADA, 2018c). Currently, some evidence however tends to discredit steroid profiles obtained from urine analyses to detect the use of exogenous AAS (Ayotte, 2010). Several confounding factors may induce high rates of false negatives. First, ranges of reference values often only refer to male Caucasian subjects that may not be extrapolated to all athletes (especially females) (Van Renterghem et al., 2010). Second, with the use of topical or transdermal formulations of T, large inter-individual variability in several markers render profile alteration only minimal or equivocal (Kotronoulas et al., 2018). Notably, low dosages of doping substances result in very short detection windows (Sottas et al., 2008a). Furthermore, the menstrual cycle undeniably impacts the ratios followed in the ABP. Changes in the T/E ratio during the cycle (due to variable excretion rates of epitestosterone) were thus reported with a marked effect of hormonal contraceptives (Schulze et al., 2014). More interestingly the use of an emergency contraceptive could potentially lead to an atypical profile in the ABP software (Mullen et al., 2017). Then, exogenous factors such as urine contamination by microorganisms (de la Torre et al., 2001; Mazzarino et al., 2011) alcohol and tea consumption (Kuuranne et al., 2014) were also reported to complicate the interpretation of steroid profiles in urine. Overall, the current urinary steroid profile in the ABP is challenged because of important pharmacological (formulation type and administration route), technical (sample preparation) and biological (bacterial, and enzymatic alteration) issues (Mareck et al., 2008).

For example, Figure 1 illustrates a clear benefit in terms of increased sensitivity (i.e., with a much lower limit of quantitation) for testosterone detection in serum vs. urine after the application of a testosterone transdermal patch. The concentration at which quantitative results can be reported with a high degree of confidence is thus much lower in blood vs. urine.

**Figure 1:**
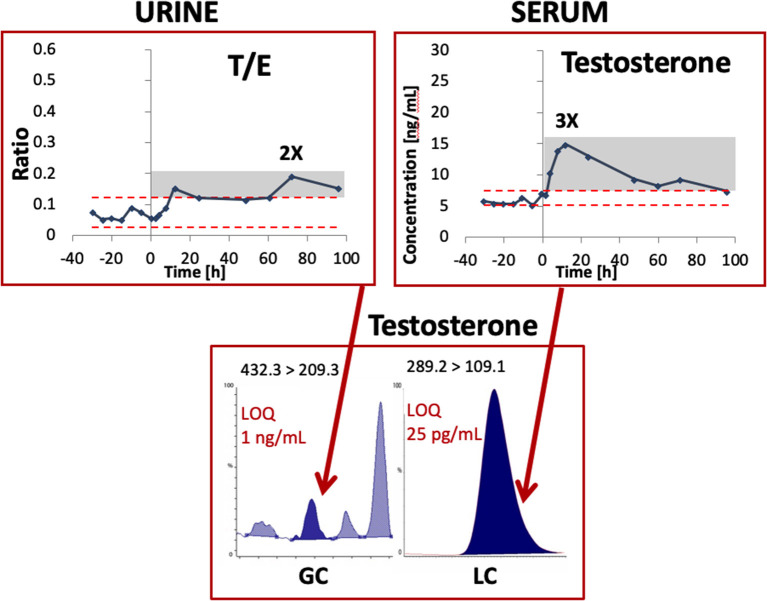
Example of testosterone detection by mass spectrometry in an urine and serum sample from one single subject after the administration of a transdermal testosterone patch. Modified from Ponzetto et al. (2018) with permission. LOQ, limit of quantitation; T/E, Testosterone/Epitestosterone ratio.

In the view of a better harmonization, WADA has enacted a technical document of sport specific analysis (WADA, 2019b) encouraging the collection of serum samples for the detection of GH use. It is thus of prime importance to highlight the good stability of the blood matrix when testing for hormones in an antidoping context. For instance, storage of serum and plasma samples at 4°C was shown to be suitable for most hormones up to 120 h (Evans et al., 2001). Similarly, in an antidoping context, insulin like growth factor-I and type III procollagen peptide were stable in serum or clotted blood samples stored at 4°C for 5 days (Holt et al., 2009). Further, in an older study, T and androstenedione were remarkably stable in plasma (with the limitation of the radioimmunoassay measurement method) (Wickings and Nieschlag, 1976). The interest of plasma samples is obvious when trying to tackle a doping scenario because doping substances are targeted unmodified in their bioactive milieu (i.e., closer to the exogenous application time), and samples are more difficult to falsify (Gosetti et al., 2013).

In terms of the chronological evolution of the main challenges and solutions in doping control analysis (Botre, 2008), state-of-the-art methods applied by accredited antidoping laboratories highlight future perspective for pertinent analyses on blood samples (Ponzetto et al., 2016). In a clinical context, medical diagnosis mostly rely on the analyses of blood samples also because of the availability of a laboratory with mass spectrometry analyses as gold standard for androgenic hormone screening (Handelsman and Wartofsky, 2013). For blood sample collected in an antidoping context (e.g., ABP samples), robust guidelines already exist to ensure limited time to analysis and sample stability (i.e., Blood Stability Score, BSS) (Robinson et al., 2016; WADA, 2019a). As a perspective to improve the ABP, the serial monitoring of steroid profiles in athletes trying to avoid AAS use detection (Alquraini and Auchus, 2018) could potentially be done from blood samples. In a recent study, an ultra-high performance liquid chromatography–high-resolution mass spectrometry (UHPLC-HRMS) method was developed for the quantification of 11 endogenous steroids in serum. In that study, concentration values measured by HRMS showed high correlation with the ones obtained by “traditional” tandem mass spectrometry (MS/MS) for all target hormones, with low absolute differences in the majority of cases suggesting that that HRMS could provide suitable performance for blood steroid analysis in the antidoping field (Ponzetto et al., 2018). In this context, “steroidomics” open the way to the untargeted simultaneous evaluation of a high number of compounds (Boccard et al., 2011). Such an approach could definitely open new antidoping perspectives for the screening of steroid metabolites after testosterone ingestion (Boccard et al., 2014) and is not limited to urinary samples. Indeed, the court of arbitration of sport has already taken a decision to sanction two female athletes because the “analysis of blood samples taken from both athletes established that such samples collected shortly before the Rio 2016 Olympic Games were found to contain an excessive concentration of testosterone” (TAS-CAS, 2019).

This decision in fact recognizes the utility of the blood analysis of steroids, because the biological interpretation of their concentration in blood, which may be affected by the intake of prohibited substances, is known to be more robust than in urine. From a legal point of view, there would be a clear advantage to use the same blood sample to “synchronize” the hematological and steroidal profiling. Repeated incentives have been formulated to improve the ABP in particular by including various information sources (Vernec, 2014) like performance data or external information from investigations.

Next-generation “omics,” especially as applied to blood samples, have long been proposed as useful markers of doping (Reichel, 2011). For example, a very robust transcriptomic response (up to 3 weeks) after recombinant human erythropoietin administration was reported (Durussel et al., 2016). At the protein and metabolite level, recent research also used steroidomics to highlight novel biomarkers of testosterone doping in serum (Ponzetto et al., 2019). Further technological progress with current initiatives (Pitsiladis et al., 2016) is thought to lead to the development of robust biomarkers that are less prone to biological and technical bias, and valid in a court of law (Neuberger et al., 2011). Since current (blood and urine) samples can be stored for up to 10 years under the current WADA Code (WADA, 2018a), it may be very useful to collect more blood samples with the future discovery of new types of target compounds in mind.

One first step could indeed be to selectively analyze hematological and steroid profile from the same blood sample. Then, the numerous serum samples collected for GH detection (as per the compulsory discipline-specific analyses (TDSSA) by WADA (WADA, 2019b) could serve as a starting point to set reference values for steroid profiles in several population types. Interestingly, such reference values (for the hematological profiles) were recently published from blood samples collected in all athletes participating in two subsequent track & field events (International Association of Athletics Federations (IAAF) World Championships) (Robinson et al., 2019). Such an ideal scenario with samples collected in an athletic cohort is however challenging and costly to conduct but the need to utilize the ABP under such conditions my help facilitate the gathering of these samples and their subsequent analysis. Finally, the rapidly increasing analytical and data processing capabilities may also open avenues for a more widespread use of dried blood spots (DBS) samples in an antidoping context (Cox and Eichner, 2017). For instance, WADA as the main regulator of antidoping policies, strategically supports advances in antidoping with methodology that uses big data, and artificial intelligence for pattern recognition (Zaier, 2014), or initiatives to use machine learning techniques to enhance detection of substances (Maass, 2019).

In conclusion, despite the limitations inherent in the use of urinary steroidal profiling described here, there remains sufficient grounds to conduct the longitudinal profiling of steroids in blood due to the recent advances in mass spectrometry. The simultaneous profiling of the hematological and steroid modules in blood may help elucidate diverse molecular pathways, and allow a more complete investigation of the proteome and the metabolome. With the prospect of enhanced detection, antidoping organizations will be compelled to utilize the full scientific potential of methods to fully exploit the stored serum samples. Successful antidoping in the future predicates further advances in the detection of prohibited substances (or methods) in plasma and serum and these developments will inevitably pave the way for more blood to be drawn from athletes.

## Author Contributions

RF and MS drafted the manuscript. RF, JS, and MS contributed to revising the manuscript and expressed their approval of the final submitted version.

### Conflict of Interest Statement

The authors declare that the research was conducted in the absence of any commercial or financial relationships that could be construed as a potential conflict of interest.
